# Peripheral iron levels in children with attention-deficit hyperactivity disorder: a systematic review and meta-analysis

**DOI:** 10.1038/s41598-017-19096-x

**Published:** 2018-01-15

**Authors:** Ping-Tao Tseng, Yu-Shian Cheng, Cheng-Fang Yen, Yen-Wen Chen, Brendon Stubbs, Paul Whiteley, Andre F. Carvalho, Dian-Jeng Li, Tien-Yu Chen, Wei-Cheng Yang, Chia-Hung Tang, Che-Sheng Chu, Wei-Chieh Yang, Hsin-Yi Liang, Ching-Kuan Wu, Pao-Yen Lin

**Affiliations:** 1 Department of Psychiatry, Tsyr-Huey Mental Hospital, Kaohsiung Jen-Ai’s Home, Kaohsiung, Taiwan; 2 WinShine Clinics in Specialty of Psychiatry, Kaohsiung, Taiwan; 3 Department of Psychiatry, Kaohsiung Medical University Hospital, Kaohsiung, Taiwan; 4 Department of Psychiatry, School of Medicine, and Graduate Institute of Medicine, College of Medicine, Kaohsiung Medical University, Kaohsiung, Taiwan; 5 Prospect clinic for otorhinolaryngology & neurology, Kaohsiung, Taiwan; 6 Physiotherapy Department, South London and Maudsley NHS Foundation Trust, London, UK; 7 Health Service and Population Research Department, Institute of Psychiatry, Psychology and Neuroscience (IoPPN), King’s College London, De Crespigny Park, London, UK; 8 Faculty of Health, Social Care and Education, Anglia Ruskin University, Chelmsford, UK; 9 ESPA Research, 2A Hylton Park Road, Sunderland, SR5 3HD UK; 10 Translational Psychiatry Research Group and Department of Clinical Medicine, Faculty of Medicine, Federal University of Ceará, Fortaleza, CE Brazil; 11 Department of Addiction Science, Kaohsiung Municipal Kai-Syuan Psychiatric Hospital, Kaohsiung, Taiwan; 12 Graduate institute of Medicine, College of Medicine, Kaohsiung Medical University, Kaohsiung, Taiwan; 13 Department of Psychiatry, Tri-Service General Hospital; School of Medicine, National Defense Medical Center Taipei, Taipei, Taiwan; 14 Department of Adult Psychiatry, Kaohsiung Municipal Kai-Syuan Psychiatric Hospital, Kaohsiung, Taiwan; 15 Department of Psychiatry, Tainan hospital, Ministry of Health and Welfare, Tainan, Taiwan; 16 Department of Psychiatry, Kaohsiung Veterans General Hospital, Kaohsiung, Taiwan; 17 Center for Geriatric and Gerontology, Kaohsiung Veterans General Hospital, Kaohsiung, Taiwan; 18 Department of pediatrics, DA-AN women and children hospital, Tainan, Taiwan; 19 Department of Child Psychiatry, Chang Gung Memorial Hospital at Taoyuan and Chang Gung University, Taoyuan, Taiwan; 20 Department of Psychiatry, Kaohsiung Chang Gung Memorial Hospital and Chang Gung University College of Medicine, Kaohsiung, Taiwan; 21 Institute for Translational Research in Biomedical Sciences, Kaohsiung Chang Gung Memorial Hospital, Kaohsiung, Taiwan

## Abstract

There is growing recognition that the risk of attention-deficit hyperactivity disorder (ADHD) in children may be influenced by micronutrient deficiencies, including iron. We conducted this meta-analysis to examine the association between ADHD and iron levels/iron deficiency (ID). We searched for the databases of the PubMed, ScienceDirect, Cochrane CENTRAL, and ClinicalTrials.gov up to August 9^th^, 2017. Primary outcomes were differences in peripheral iron levels in children with ADHD versus healthy controls (HCs) and the severity of ADHD symptoms in children with/without ID (Hedges’ *g*) and the pooled adjusted odds ratio (OR) of the association between ADHD and ID. Overall, seventeen articles met the inclusion criteria. Peripheral serum ferritin levels were significantly lower in ADHD children (children with ADHD = 1560, HCs = 4691, Hedges’ *g* = −0.246, *p* = 0.013), but no significant difference in serum iron or transferrin levels. In addition, the severity of ADHD was significantly higher in the children with ID than those without ID (with ID = 79, without ID = 76, Hedges’ *g* = 0.888, *p* = 0.002), and there was a significant association between ADHD and ID (OR = 1.636, *p* = 0.031). Our results suggest that ADHD is associated with lower serum ferritin levels and ID. Future longitudinal studies are required to confirm these associations and to elucidate potential mechanisms.

## Introduction

Attention-deficit hyperactivity disorder (ADHD) is a common neurodevelopmental condition with an estimated global prevalence of 7.2%^1^. ADHD can cause considerable disability and impaired quality of life^2^. Furthermore, children with ADHD also suffer from a high prevalence of co-occurring medical and psychiatric conditions such as depressive disorder, anxiety disorder, restless leg syndrome, enuresis, and serious stomach problems^3^.

ADHD is currently thought to be a predominantly heritable condition, with genetic polymorphisms of dopamine receptors and dopamine transporters playing an important role^4^. While the pathophysiology of ADHD is still not entirely understood, dysregulated dopaminergic neuronal development in the prefrontal cortex and subcortical regions is believed to play a role based on research derived from neuroimaging studies and the effects of pharmacotherapeutic treatment^5^. Recently, the potential effect of environmental (non-genetic) factors has attracted attention in the etiology of ADHD^6^. Environmental factors are potentially modifiable and may represent preventable targets for these disorders, in addition to potentially providing novel therapies for certain aspects of ADHD, such as iron supplementation^7^. Among environmental risk factors, nutritional factors including certain micronutrient deficiencies have been increasingly implicated as possible risk factors for ADHD^8^.

Iron helps in homeostasis of the hemoglobin structure, antioxidants, genetic repair, and in particular central nervous system (CNS) function^9^. Iron is stored safely in the body via binding to ferritin, which prevents the degrading effects of free iron. Iron is very important for early brain development and is required for normal myelination and neurotransmitter function. Evidence has shown that impaired iron absorption and subsequent iron deficiency (ID) in both the prenatal and postnatal periods may impair neurodevelopment, with long-lasting and possibly irreversible consequences^10^. In addition, ID may result in myelinating and/or synaptogenesis dysregulation^11^. Moreover, ID may be associated with impaired monoamine synthesis, resulting in dysregulated monoamine signal transduction^10,12^. Adequate iron intake and peripheral iron levels may therefore be important factors modifying the onset of ADHD or ameliorating the severity of ADHD^13^.

To date, three meta-analyses have evaluated peripheral iron levels, either in forms of iron or ferritin, in children with ADHD^14–16^. While helpful, two of these meta-analyses only considered peripheral ferritin levels. This limitation may have resulted in confounding effects, since peripheral ferritin levels can be affected by various factors including infection, inflammation, and oxidative stress and, therefore, may not represent the whole picture of iron storage in clinical practice^17^. In the most recent meta-analysis, Wang *et al*. updated the evidence of the relationship between iron and ADHD, and revealed significantly lower serum ferritin levels in children with ADHD than in controls (standardized mean difference (SMD) = −0.40, 95% CI = −0.66 to −0.14) but not serum iron (SMD = −0.026, 95% CI = −0.29 to 0.24)^16^. Based on this insignificant result of serum iron in ADHD and controls, the relationship between iron and ADHD still remained unclear. Furthermore, that recent meta-analysis did not provide information about other parameters of iron status such as transferrin level^16^. Furthermore, no previous study has investigated the relationship between the diagnosis of ID and the risk or severity of ADHD. Although the use of medications for ADHD has been well-established, certain side effects such as poor appetite may still limit their use^18^. Several studies have investigated the use of iron supplements for patients with ADHD with mixed results, however none of these studies specifically targeted patients with a diagnosis of ID^13,19^. In addition, two previous meta-analyses found lower ferritin level in patients with ADHD, however it is unclear whether such lower ferritin levels in patients with ADHD reaches a clinical diagnosis of ID^14,15^. Therefore, investigating the relationship between a diagnosis of ID and the risk and severity of ADHD may help to identify potential treatments such as iron supplementation.

The aim of the current study was to conduct an updated systematic review and meta-analysis considering differences in peripheral iron levels and all parameters of iron status between children with ADHD and healthy (asymptomatic) controls (HCs). In addition, we also aimed to explore the association between a diagnosis of ID and the risk and severity of ADHD.

## Results

### Study selection

**Figure 1:**
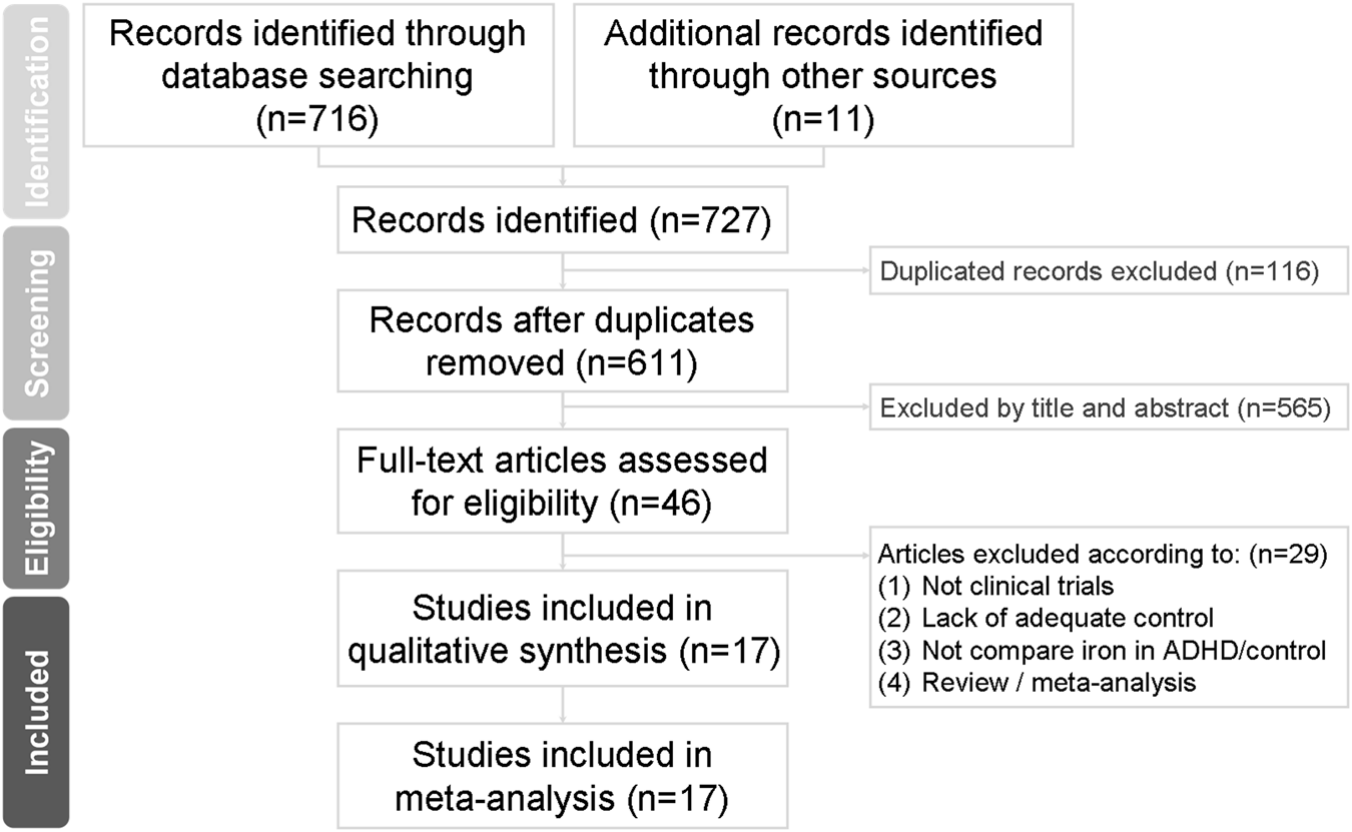
Flowchart of the selection strategy and inclusion/exclusion criteria for the current meta-analysis.

Figure 1 summarizes the details of the search results. In brief, a total of 46 studies entered the full-text review stage. Twenty-nine articles were excluded for various reasons including a lack of controls, non-clinical trials, not comparing iron between ADHD/controls, or review articles (see Supplementary Table 2). A list of excluded articles is presented in Supplementary Table 2. In total, twenty-two articles met the inclusion criteria. We were unable to conduct meta-analysis for four of the recruited studies, because we did not have enough studies (n < 3) for the outcome measurements such as hair iron level, food iron intake level, or plasma/blood iron level in children with ADHD versus controls, or the prevalence of ADHD in children with and without ID^20–23^. Finally, we recruited seventeen studies in our meta-analysis^9,24–39^, and the details are summarized in Table 1.

**Table 1:** Summary of included studies comparing comorbidity of iron deficiency and ADHD.

Author (year)	Dx Criteria	sample source	Assay iron detection	Groups	Subject numbers	Comparison	Primary outcome	Mean age in subgroup	Female (%) in subgroup
*Subgroup meta-analysis of comparison of the peripheral iron levels in children with ADHD and those without ADHD (Study number=18)*									
Bala, K.A.^25^	DSM-IV	serum	CI method	ADHD HC	34 27	serum ferritin	39.4 ± 15.0 29.1 ± 17.7	7.7 ± 3.2 9.8 ± 4.0	32.4 51.9
Percinel, I.^38^	DSM-IV	serum	n/a	ADHD HC	200 100	serum iron serum ferritin	(ug/dl) (iron)71.9 ± 31.0 77.9 ± 30.6 (ng/ml) (ferritin)27.9 ± 15.3 30.8 ± 17.5	11.0 ± 2.4 11.0 ± 3.0	36.5 40.0
Adisetiyo, V.^51^	DSM-IV	serum	n/a	ADHD medicated ADHD no medicated HC	10 12 27	serum iron serum ferritin	(serum iron) 79.5 ± 23.3 74.8 ± 27.9 65.7 ± 28.2 (serum ferritin) 50.3 ± 26.1 51.3 ± 24.1 38.2 ± 22.8	13.5 ± 2.2 11.9 ± 3.1 13.3 ± 2.6	30.0 33.3 55.6
Bener, A.^26^	DSM-IV	serum	RIA	ADHD HC	630 630	serum iron serum ferritin	(ug/dl) (iron)82.1 ± 13.6 85.6 ± 12.5 (ng/ml) (ferritin)36.3 ± 5.9 38.2 ± 5.6	11.5 ± 3.8 11.5 ± 3.6	50.0 50.3
Donfrancesco, R.^29^	DSM-IV	serum	ELISA	ADHD HC	101 93	serum ferritin	(ng/ml)33.0 ± 17.8 33.1 ± 18.7	8.9 ± 2.5 9.2 ± 3.1	8.9 11.8
Romanos, M.^39^	n/a	serum	n/a	Abnormal SDQ^&^ Normal SDQ^&^	240 2565	serum ferritin	(ng/ml)46.6 ± 21.5 45.2 ± 21.5	10.0 ± 0.2	49.1
Cortese,^28^	DSM-IV	serum	Fer.	ADHD HC	18 9	serum ferritin	(ng/ml)32.4 ± 13.4 51.6 ± 16.4	9.9 ± 1.5 10.1 ± 2.2	11.1 44.4
Kwon, H.J.^35^	DSM-IV	serum	n/a	ADHD HC	48 48	serum iron serum ferritin serum transferrin	(ug/dl) (iron)80.9 ± 33.3 82.0 ± 28.1 (ng/ml) (ferritin)35.8 ± 16.6 37.1 ± 18.3 (transferrin)(mg/dl) 248.4 ± 44.2 266.3 ± 25.4	7.5 ± 0.6 7.5 ± 0.6	46.5 46.5
Juneja, M.^32^	DSM-IV	serum	ELISA	ADHD HC	25 25	serum ferritin	(ng/ml)6.0 ± 3.9 49.0 ± 41.6	8.4 ± 1.7 8.0 ± 1.5	16.0 16.0
Menegassi M.^36^ (+Mx)	DSM-IV	serum	Fer.	ADHD + MP Controls	19 21	serum iron serum ferritin serum transferrin	(ug/dl) (iron)80.6 ± 30.3 92.0 ± 31.4 (ng/ml) (ferritin)59.3 ± 21.0 58.8 ± 28.9 (mg/dl) (transferrin)270.9 ± 25.9 267.1 ± 29.3	8.8 ± 2.4 8.9 ± 2.7	21.1 28.6
Menegassi,^33^ (no Mx)	DSM-IV	serum	Fer.	ADHD without Mx Controls	22 21	serum iron serum ferritin serum transferrin	(ug/dl) (iron)78.6 ± 24.0 92.0 ± 31.4 (ng/ml) (ferritin)54.2 ± 17.2 58.8 ± 28.9 (mg/dl) (transferrin)253.4 ± 24.4 267.1 ± 29.3	9.0 ± 2.6 8.9 ± 2.7	27.3 28.6
Konofal, E.^33^ (+RLS)	DSM-IV	serum	n/a	ADHD + RLS HC	12 10	serum ferritin	(ug/l)16.0 ± 6.0 46.0 ± 18.0	7.3 ± 1.2 7.0 ± 0.9	33.3 30.0
Konofal, E.^37^ (no RLS)	DSM-IV	serum	n/a	ADHD HC	10 10	serum ferritin	(ug/l)25.0 ± 15.0 46.0 ± 18.0	6.7 ± 0.9 7.0 ± 0.9	10.0 30.0
Millichap, J.G. (2006)	n/a	serum	n/a	ADHD HC	68 1053	serum ferritin	(ng/ml)39.9 ± 40.6 37.5 ± 49.0	n/a	20.6 n/a
Chen, J.R.^27^ serum	DSM-IV	serum	n/a	ADHD HC	58 52	serum iron	(umol/L)19.7 ± 6.4 16.1 ± 6.8	8.5 ± 2.2 7.9 ± 2.0	25.9 23.1
Konofal, E.^34^	DSM-IV	serum	ELISA	ADHD HC	53 27	serum ferritin	(ng/ml)23.0 ± 13.0 44.0 ± 22.0	n/a	15.1 25.9
*Subgroup meta-analysis of comparison of the symptoms severity of ADHD in children with iron deficiency and those without iron deficiency (Study number = 7)*									
Doom, J.R.^30^	n/a	serum	n/a	IDA^%^ No anemia^%^	7 50	Scores of ADHD symptoms	0.8 ± 1.0 −0.03 ± 0.6	5.0 5.0	n/a
Bener, A.^26^	DSM-IV	serum	RIA	total	(total) 1260	ADHD odds ratio	(iron deficiency) odds ratio=2.8 (95% CI: 1.7-4.5) (ferritin) odds ratio=2.5 (95% CI: 1.8-3.5)	11.5 ± 3.7	50.2
Abou-Khadra, M.K.^24^	DSM-IV	serum	ELISA	Ferritin < 30 ng/ml Ferritin > 30 ng/ml	25 16	CPRS-RL ADHD index	26.7 ± 5.5 24.7 ± 4.3	8.0 ± 1.7	14.6
Chen, M.H.^9^	ICD-9	diagnosis	n/a	IDA HC	2957 11828	ADHD diagnosis	84 (2.8%) 207 (1.8%)	10.6 ± 6.0 10.6 ± 6.0	64.2 64.2
Fuglestad, A.J.^31^	n/a	blood	n/a	IDA HC	47 10	TBAQ-R^$^	3.9 ± 0.7 4.5 ± 0.9	1.7 ± 0.8 1.4 ± 0.3	59.6 40.0
Romanos, M.^39^	n/a	serum	n/a	Children total	2805	SDQ^&^	Odds ratio = 1.1 (95% CI: 0.9 - 1.3)	10.0 ± 0.2	49.1

*Derived effect sizes from other data, such as sample size and p value. ^&^Used outcome from SDQ hyperactivities/inattention scores. ^%^Sample from international adoption data. ^$^TBAQ-R activity and impulsivity scores. Abbreviation: ADHD: Attention deficit hyperactivity disorder; ADHD-RS: ADHD rating scale; CI: confidence interval; CI method: Chemiluminescent method; CPRS: Conners’ parents rating scales; CTRS: Conners’ teacher rating scales; DSM-III: diagnostic and statistical manual of mental disorders, third edition; DSM-IV: diagnostic and statistical manual of mental disorders, fourth edition; Dx: diagnosis; Fer.: Ferrozine method; HC: health control; ICD-9: international statistical classification of diseases and related health problems 9th revision; ID: iron deficiency;

IDA: iron deficiency anemia; MA: meta-analysis; Mx: medication; MP: methylphenidate; n/a: not available; OR: odds ratio; PDD: pervasive developmental disorder; RIA: Radio-immunoassay; RLS: restless leg syndrome; SDQ: Strengths and difficulties questionnaire; TBAQ-R: Toddler behavior assessment questionnaire-revised; Tx: treatment. Based on the hypothesis of normal distribution of the peripheral iron levels and prevalence of ADHD, we merged the different outcomes from recruited studies into one single outcome, the Hedges’ G. (Reference: Hedges LV: Statistical considerations. In: The Handbook of research synthesis and meta-analysis. 2nd edn. Edited by COOPER H, Hedges LV, Valentine JC. New York: Russell SAGE foundation; 2009: 38–46).

### Characteristics and methodological quality of the included studies

Of the included studies, thirteen provided information about serum ferritin levels, six about serum iron levels, two about serum transferrin levels, three about the severity of ADHD symptoms in patients with ID compared to controls, and three provided data of adjusted odds ratios (ORs) with regards the association between ADHD and ID.

We were unable to conduct meta-analysis for the prevalence of ADHD in children with and without ID because we did not have enough studies (n < 3)^9^. Rather, we chose differences in the severity of ADHD symptoms in children with and without ID to indicate the presence of ADHD.

#### Study quality appraisal

Regarding the methodological quality of the included studies, the average modified Newcastle-Ottawa Scale (NOS) score^40^ for case-control trials in subgroup meta-analysis of comparisons of the peripheral iron levels in children with and without ADHD was 6.82 (high quality) with a standard deviation (SD) of 1.17^41^. The NOS score for cross-sectional trials in subgroup meta-analysis of comparisons of the peripheral iron levels in children with and without ADHD was 4.67 (high quality) (SD = 0.58)^41^. In addition, the average modified NOS scores for cohort trials and cross-sectional trials in subgroup meta-analysis of comparisons of the severity of ADHD symptoms in children with and without iron deficiency were 5.00 (low quality) (SD = 1.41)^40^ and 5.75 (high quality) (SD = 2.50)^41^, respectively (Supplementary Table 3).

### Meta-analysis of peripheral iron levels in the children with ADHD versus controls: peripheral ferritin

**Figure 2:**
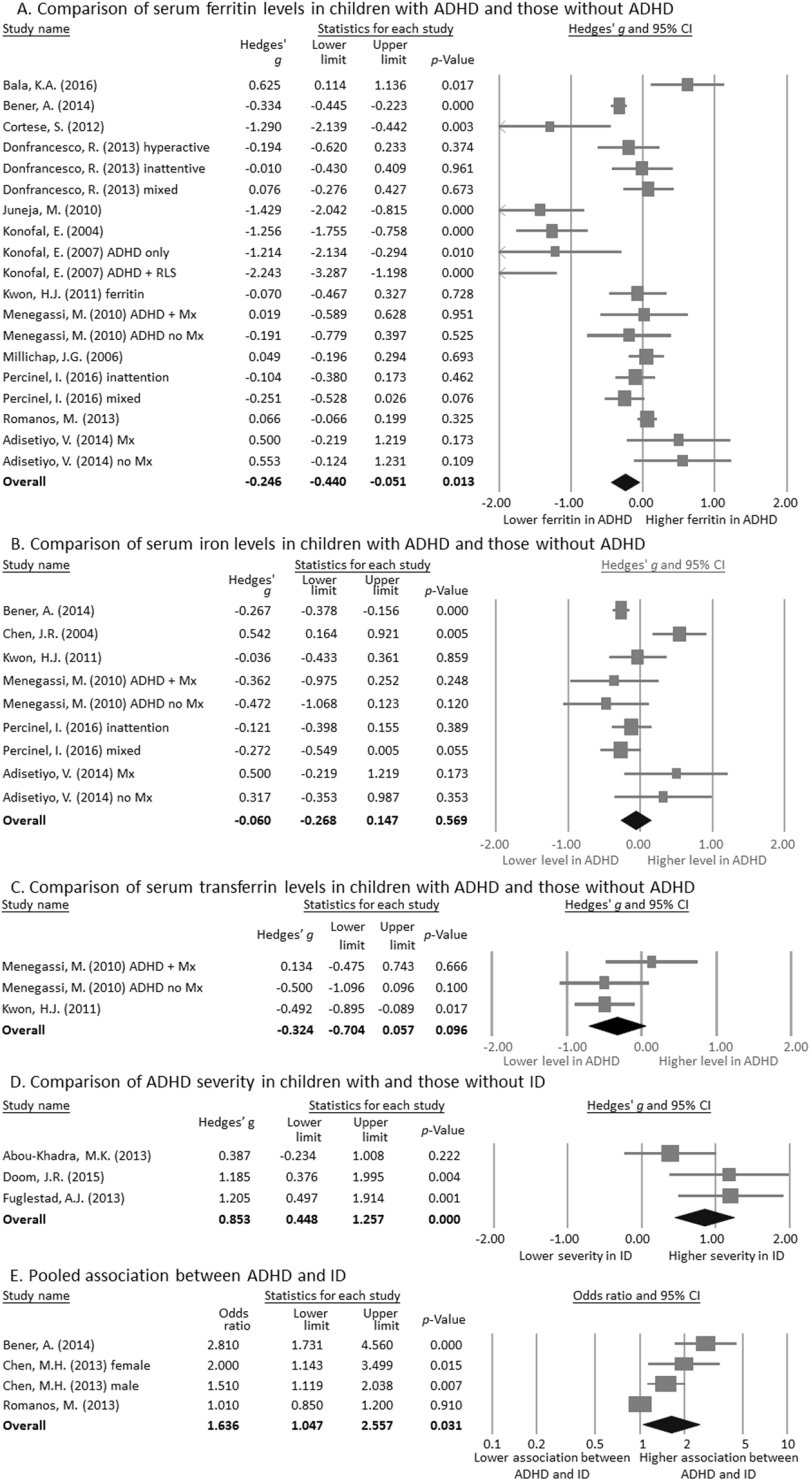
Forest plots showing effect sizes (Hedges’ *g*) and 95% confidence intervals (CIs) from individual studies and pooled results of all included studies comparing (**A**) serum ferritin, (**B**) serum iron, and (**C**) serum transferrin levels in children with and without ADHD; (**D**) Forest plot showing effect sizes (Hedges’ *g*) and 95% CIs from individual studies and pooled results comparing the severity of ADHD symptoms in children with and without iron deficiency (ID); (**E**) Forest plot pooling the adjusted odds ratio (OR) comparing the association between ADHD and ID. Figure 2(A) serum ferritin levels (*p* = 0.013) but not (**B**) serum iron (*p* = 0.569) or (**C**) serum transferrin (*p* = 0.096) levels were significantly lower in the children with ADHD compared to those without ADHD. Figure 2(D) The severity of ADHD symptoms was significantly greater in the children with ID than in those without ID (*p* < 0.001). Figure 2(E) indicated a significantly high association between ADHD and ID based on the pooled adjusted OR (*p* = 0.031).

Overall, the meta-analysis results demonstrated that the peripheral serum ferritin level in the children with ADHD (n = 1560, mean age = 10.1, mean female proportion = 36.6%) was significantly lower than in the children without ADHD (n = 4691, mean age = 10.4, mean female proportion = 21.9%)^25,26,28,29,32–39,42^ (*k* = 19, Hedges’ *g* = −0.246, 95% confidence interval (CI) = −0.440 to −0.051, *p* = 0.013) (Fig. 2A). There was evidence of high heterogeneity (Q value = 105.528, df = 18, *I*^2^ = 82.94%, *p* < 0.001) but not publication bias according to Egger’s regression test (t = 0.816, df = 17, *p* = 0.426). The significance of the results did not change after sensitivity analysis with the removal of one study. However, meta-analysis could not be performed on plasma ferritin or peripheral blood ferritin levels because there were fewer than three datasets.

#### Meta-regression analysis

The ESs of differences in serum ferritin levels between the children with ADHD and controls had significantly positive association between mean age (slope = 0.187, *k* = 17, *p* = 0.025) but not significantly moderated by proportion of females (*p* = 0.061), percentage of combined subtypes of ADHD (*p* = 0.550), percentage of the inattention subtype of ADHD (*p* = 0.860), percentage of the hyperactivity/impulsivity subtype of ADHD (*p* = 0.884), cognition (in forms of mean IQ) (*p* = 0.574), sample size of the group (*p* = 0.740), and geographical latitude of where the study was conducted (*p* = 0.690) (Supplementary Table 4).

#### Subgroup meta-analysis

To identify potential sources of heterogeneity, we focused on the studies recruiting children who did not receive medications. Three studies with four datasets were included^34,38,42^, and the results of meta-analysis showed that there was no significant difference in serum ferritin level between the children with ADHD and the controls (*k* = 4, Hedges’ *g* = −0.287, 95% CI = −0.812 to 0.237, *p* = 0.283).

### Meta-analysis of peripheral iron levels in the children with ADHD versus controls: peripheral iron

Meta-analysis demonstrated that the serum iron level in children with ADHD (n = 941, mean age = 11.1, mean female proportion = 45.4%) was not significantly different from the children without ADHD (n = 847, mean age = 11.2, mean female proportion = 48.0%)^26,27,35,36,38,42^ (*k* = 9, Hedges’ *g* = −0.060, 95% CI = −0.268 to 0.147, *p* = 0.569) (Fig. 2B). There was evidence of high heterogeneity (Q value = 24.271, df = 8, *I*^2^ = 67.04%, *p* = 0.002) but no significant publication bias according to the funnel plot (Supplement Fig. 2). After removing the study by Chen (2004) via sensitivity testing, the pooled data suggested that there was a significantly lower serum iron level in the children with ADHD compared to the controls (Hedges’ *g* = −0.186, 95% CI = −0.323 to −0.048, *p* = 0.008)^27^.

#### Meta-regression analysis

The ESs of differences in serum iron levels between the children with ADHD and controls were not significantly moderated by mean age (*p* = 0.827), proportion of females (*p* = 0.958), sample size of the ADHD group (*p* = 0.388), and geographical latitude of where the study was conducted (*p* = 0.361) (Supplementary Table 4).

### Meta-analysis of peripheral iron levels in the children with ADHD versus controls: peripheral transferrin

Meta-analysis demonstrated that there was no significant difference in serum transferrin level between the children with (n = 89, mean age = 8.2, mean female proportion = 36.3%) and without ADHD (n = 90, mean age = 8.2, mean female proportion = 38.1%)^35,36^ (*k* = 3, Hedges’ *g* = −0.324, 95% CI = −0.704 to 0.057, *p* = 0.096) (Fig. 2C). There was no evidence of significant heterogeneity (Q value = 3.147, df = 2, *I*^2^ = 36.440%, *p* = 0.207) or publication bias according to the funnel plot (Supplementary Figure 2). In the sensitivity test, the insignificant meta-analysis result became significant, and the children with ADHD had a significantly lower level of serum transferrin than those without ADHD after removing the subjects who received methylphenidate in the study by Menegassi, M. (2010) (Hedges’ *g = *−0.494, 95% CI = −0.828 to −0.160, *p* = 0.004)^36^. We could not perform meta-analysis on plasma transferrin or peripheral blood transferrin levels because there were fewer than three datasets.

#### Meta-regression analysis

Meta-regression and subgroup meta-analysis could not be performed as fewer than the minimal number of datasets were available.

### Meta-analysis of the severity of ADHD in the children with ID versus controls

The severity of ADHD symptoms was significantly higher in the children with ID than in those without ID^24,30,31^ (79 children with ID, 76 children without ID, total mean age = 4.6, total mean female proportion = 38.7%) (*k* = 3; Hedges’ *g* = 0.888, 95% CI = 0.327 to 1.450, *p* = 0.002) (Fig. 2D) without evidence of heterogeneity (Q value = 3.763, df = 2, *I*^2^ = 46.850%, *p* = 0.152) or publication bias via inspection of the funnel plot (Supplementary Figure 2). The sensitivity test revealed that the significant meta-analysis result became insignificant after removing the studies either by Doom, J. R. (2015) (Hedges’ *g* = 0.777, 95% CI = −0.024 to 1.579, *p* = 0.057)^30^ or Fuglestad, A. J. (2013) (Hedges’ *g* = 0.742, 95% CI = −0.036 to 1.520, *p* = 0.062)^31^.

#### Meta-regression analysis

Meta-regression and subgroup meta-analysis could not be performed as fewer than the minimal number of datasets were available.

### Meta-analysis of the association between ADHD and ID: the pooled adjusted OR

The meta-analysis of the pooled adjusted OR for the association between ADHD and ID revealed a significant association between ADHD and ID^9,26,39^ (*k* = 4; OR = 1.636, 95% CI = 1.047 to 2.557, *p* = 0.031) (Fig. 2E) with significant evidence of heterogeneity (Q value = 20.861, df = 3, *I*^2^ = 85.619%, *p* < 0.001) and publication bias via inspection of the funnel plot (Supplementary Figure 2). The adjusted ESs with publication bias through Duval and Tweedie’s trim and fill test were insignificant (OR = 1.217, 95% CI = 0.812 to 1.824). Furthermore, the sensitivity test revealed that the significant meta-analysis result became insignificant after removing the study by Bener, A. (2014) (OR = 1.364, 95% CI = 0.928 to 2.005, *p* = 0.114)^26^, the female subjects in the study by Chen, M. H. (2013) (OR = 1.556, 95% CI = 0.928 to 2.609, *p* = 0.093)^9^, or the male subjects in the study by Chen, M. H. (2013) (OR = 1.732, 95% CI = 0.855 to 3.506, *p* = 0.127)^9^. Meta-regression and subgroup meta-analysis could not be performed as fewer than the minimal number of datasets were available.

## Discussion

The main results of our review and meta-analysis suggest that serum ferritin levels, but not iron or transferrin levels, are significantly lower in children diagnosed with ADHD than in those without ADHD. However, when removal of one potential confounding study, the result of meta-analysis of serum iron changed into significantly lower serum iron level in the children with ADHD compared to the controls. Children with ID were also more likely to have ADHD and have more severe ADHD symptoms than those without ID.

Our results are in general agreement with three previous meta-analyses^14–16^ (Table 2). However, our findings not simply confirm the same results in previous reports but also added further information upon current scientific knowledge, such as a higher odds of ADHD and higher severity of ADHD symptoms in the patients with ID and significantly lower serum iron level in the children with ADHD compared to the controls in specific post-hoc meta-analysis, such as sensitivity testing. Specifically, we found that the ORs and symptom severity of ADHD were higher in the patients with ID. Together with a lower ferritin level, this result may suggest an association between a lower serum iron level and ADHD symptomatology. Moreover, although both our meta-analysis and most recent meta-analysis by Wang *et al*. showed no significant difference in serum iron levels between ADHD and control groups^16^, sensitivity test was not done in the study by Wang *et al*. After we re-investigated the potential sources of insignificant results through sensitivity test (Table 2), we found significantly lower serum iron levels in the children with ADHD compared to those without ADHD after removing the study by Chen *et al*.^27^. In the study by Chen and colleagues (2004), we identified several significantly different baseline variables between the ADHD and control groups, including higher food iron intake and higher food vitamin C intake in the ADHD group. Vitamin C is known to enhance iron absorption from food^43^. Knowing that human iron nutrients primarily come from food intake, the children with ADHD included in the study by Chen who had significantly higher iron and vitamin C intake would be expected to show elevated blood iron levels. Such information about food intake was not provided by the other studies included in our subgroup meta-analysis of serum iron levels^26,35,36,38^. Therefore, it was reasonable to exclude data from the study by Chen *et al*.^27^ when interpreting the results of subgroup meta-analysis of serum iron levels. Following exclusion from this study, the results of subgroup meta-analysis of serum iron levels revealed a significantly lower serum iron level in the children with ADHD than in those without ADHD (Hedges’ *g* = −0.186, 95% CI = −0.323 to −0.048, *p* = 0.008). This provides further evidence of a shortage of body iron stores in children diagnosed with ADHD. Taken together, our findings provide tentative evidence that deficient iron storage in children with ADHD may be involved in the pathophysiology of the condition. However, future longitudinal research is required to confirm/refute this tentative hypothesis.

**Table 2:** Summary and comparison of main findings of meta-analysis about peripheral iron levels in ADHD.

Article	Search date	Study numbers	Target iron in ADHD	Primary outcome	Meta-regression
Tseng PT (2017) (current MA)	May 1, 2017	22	Serum iron Serum ferritin Serum transferrin	No different serum iron between ADHD and control (Hedges’ *g* = −0.060, 95% CI = −0.268 to 0.147, *p* = 0.569) (HE: *p* = 0.002, PB: not significant) but changed to significant **lower** serum **iron** in ADHD after removal potential confounding one study (Hedges’ *g* = −0.186, 95% CI = −0.323 to −0.048, *p* = 0.008) **Lower** serum **ferritin** in ADHD than control (Hedges’ *g* = −0.246, 95% CI = −0.440 to −0.051, *p* = 0.013) (HE: *p* < 0.001, PB: *p* = 0.426) No different serum transferrin between ADHD and control (Hedges’ *g* = −0.324, 95% CI = −0.704–0.057, *p* = 0.096), HE: *p* = 0.207, PB: *p* = 0.587	None of the clinical variables has association
Wang Y.^16^	Jul 25, 2016	11	Serum ferritin Serum iron	**Lower** serum **ferritin** in ADHD than control (SMD = −0.40, 95% CI = −0.66 to −0.14) No difference in serum iron (SMD = −0.026, 95% CI = −0.29 to 0.24)	None of the clinical variables has association
Scassellati C.^14^	Sep, 2011	7	Blood ferritin	**Lower** blood **ferritin** in ADHD than control (d = −0.86, Z = 2.52, *p* = 0.01), HE: *p* < 0.001	n/d
Tan LN.^15^	Mar, 2011	5	Blood ferritin	**Lower** blood **ferritin** in ADHD than control (mean difference = −23.09, 95% CI = −33.06 to −13.13, Z = 4.54, *p* < 0.001), HE: *p* = 0.003, PB: N.S.	n/d

Abbreviation: ADHD: Attention deficit hyperactivity disorder; ASD: autistic spectrum disorder; CI: confidence interval; HE: heterogeneity; n/d: not done; ID: iron deficiency; N.S.: not significant; OR: odds ratio; PB: publication bias; SMD: standardized mean difference.

The relationship between ID and ADHD may be explained by several possible pathophysiological mechanisms. First, low peripheral iron levels, indicating insufficient iron storage, may dysregulate dopaminergic neurons, which may play a prominent role in the pathoetiology of ADHD^5,44,45^. In brief, iron aids in dopamine synthesis by acting as a co-factor for tyrosine hydroxylase, which is a rate-limiting enzyme for the conversion of hydroxylation of tyrosine to L-DOPA, a precursor of dopamine^46^. Iron deficiency may therefore result in disruption of dopamine activity, as shown in several animal studies^12,47,48^. This dysregulation of dopaminergic neurons may further result in multiple frontal dysfunctions that mimic the symptoms of ADHD^49^. In addition, ADHD has been found to be more prevalent in patients with restless leg syndrome (RLS) (27.62% with a diagnosis of ADHD), and iron deficiency and dopamine system dysregulation has also been reported to play an important role in RLS^47,48,50^. Therefore, iron deficiency with resulting brain dopamine dysfunction may be a common pathway for the pathophysiology of both disorders. In addition to the dopamine theory, lower ferritin levels may provide indirect evidence of elevated oxidative stress^17^, and heightened oxidative stress has also been reported in patients with ADHD^51^. This increased oxidative stress burden may disturb neurodevelopmental trajectories and gene functions potentially predisposing to the onset of ADHD^51^. However, given the observational nature of our data, the precise mechanisms and directionality of any relationships we observed cannot be verified.

Our meta-analysis focused on peripheral iron levels rather than brain iron levels. The extent to which peripheral and brain iron levels are correlated remains unclear. Cortese and colleagues provided some evidence that brain iron levels in the bilateral thalamus were significantly lower in children with ADHD compared to healthy controls^28^. However, another MRI study by Adisetiyo *et al*. failed to find an association between brain iron levels and serum ferritin levels^42^. Therefore, further studies are required to explore the relationship between brain and serum iron levels.

In the main result of our meta-analysis, high heterogeneity had been detected through the results. To address the potential source of heterogeneity, we arranged subgroup meta-analysis and meta-regression to investigate it. In part of subgroup analysis, if we only included the studies that recruited children who did not take medications, the difference in ferritin level between the ADHD and control groups became non-significant. Although, no obvious interactions have been reported between most drugs and ferritin level, D’Amato suggested that methylphenidate may cause a poor appetite and possibly less iron intake in children with ADHD^52^. However, only four of the nineteen datasets recruited drug-free participants, and most of the other studies did not provide much information about the medications prescribed to their participants. Therefore, further studies are warranted including drug-free participants only to further investigate the relationship between methylphenidate and ferritin level in children with ADHD. In addition to the potentially confounding effect by medication, the different food intake pattern would also contribute impact on the iron storage. For example, Lane and the colleague (2014) provided evidences about the enhancing effect on iron absorption by vitamin C intake^43^. Studies recruited subjects with high vitamin C intake or iron supplementation would have confounded results compared to others^27^.

Finally, in part of meta-regression, we tried to address the potential impact of some clinical variables on the peripheral iron levels. The differences in serum ferritin levels between the children with ADHD and the controls were only significantly moderated by mean age but not by other factors including proportion of females, subtype of ADHD, cognition (in forms of mean IQ), sample size of the ADHD group, and geographical latitude of where the study was conducted. We are uncertain about the significance of the finding that the difference in ferritin level in ADHD and control groups became larger with age because there were no previous reports or studies addressed this issue. However, previous studies showed that the reference range of ferritin level became wider and higher with age in pediatric population^53^. Therefore, further studies investigating ferritin level and ADHD symptoms may need to take this factor into account, before analyzing their study results. On the other hand, our meta-regression did not show significant associations between the prevalence of ADHD in children with ID and mean age, mean proportion of females, and geographic latitude of where the study was conducted.

## Limitations

There are several limitations to the current study. First, the total number of included studies was relatively small, and therefore there was a risk of type I and type II errors. Second, our main targets focused on peripheral samples of iron status parameters rather than CNS parameters because few studies provided such information^28,42^. Third, we could not fully exclude the potential confounding effect of food iron intake on peripheral iron status because only a few studies provided such information^27^. In addition, we could not completely rule out the potential confounding effect of the ability of different types of assay to detect iron due to the inconsistent reporting of those data across the included studies. Fourth, we lacked comparisons of changes in iron status in the children with ADHD in long-term follow-up because there were few cohort studies^7,39^. Fifth, we could not perform subgroup meta-analysis of plasma ferritin, iron, or transferrin due to the limited number of datasets available in the studies. Finally, we could not perform meta-regression of peripheral iron levels and attention because of the limited data available.

## Conclusions

The results of our meta-analysis suggest that children diagnosed with ADHD have lower serum ferritin levels compared to those without ADHD. We also observed that the children with ID were more likely to have ADHD and to suffer from more severe ADHD symptoms compared to those without ID. We therefore suggest that further studies are warranted to explore the benefits of iron supplementation in children with ADHD with ID, in particular those with more severe ADHD symptoms. However, given the cross-sectional nature of most of the available studies, further longitudinal and cohort studies are required to thoroughly evaluate the relationships between iron status and ADHD symptoms, and to elucidate the potential pathophysiological mechanisms. In addition, further studies may be needed to investigate the relationship between methylphenidate and ferritin level to exclude any potential effects of methylphenidate on oral iron intake.

## Methods and Materials

The current study followed the Meta-analysis Of Observational Studies in Epidemiology (MOOSE) guidelines^54^ (see Supplementary Table 1 and Supplementary Figure 1). The meta-analysis followed our previous defined but unpublished protocol and was approved by the Institutional Review Board of Tri-Service General Hospital (TSGHIRB: B-105-12).

### Eligibility criteria

The inclusion criteria were: (a) observational studies, including a cohort or cross-sectional study design, comparing all peripheral iron status parameters including iron, ferritin, and transferrin levels in children with ADHD (confirmed by either a structured or non-structured diagnostic interview) and controls; and (b) clinical human studies. We excluded preclinical studies, review articles, meeting abstracts, or peer-reviewed original articles not conducted in humans.

### Search strategy and study selection

Two independent authors conducted an electronic systematic literature search from inception to August 9^th^, 2017 across PubMed, ScienceDirect, Cochrane CENTRAL, and ClinicalTrials.gov databases. We used the following keywords: “(iron OR ferritin OR ferrous) AND (Attention deficit hyperactivity disorder OR ADHD)”. To expand our eligible list, we consulted the reference lists of the included articles and recent reviews^14,55,56^.

At the eligibility stage, two authors (YS Cheng and PT Tseng) screened the titles and abstracts of all results to assess potential eligibility. The authors then reviewed the full-text articles which were deemed potentially eligible. A final list of included studies was determined and any inconsistencies were resolved by consensus or via thorough discussion with a third reviewer (PY Lin).

### Primary outcomes

We set the primary outcomes as the difference in peripheral iron levels (including iron, ferritin, or transferrin) between children with ADHD and controls, and the OR of ADHD or severity of ADHD symptoms between children with or without ID. If the data of interest were not available in the articles, we contacted the authors twice over a month to request the data.

### Data extraction

Two independent authors (YS Cheng and PT Tseng) extracted data using a predetermined list of variables of interest, which included: prevalence/incidence rates of ADHD, prevalence rates of iron deficiency, peripheral iron levels, amount of food iron intake, mean age, gender distribution in the form of the percentage of females, body mass index, percentage of ADHD subtypes, cognitive performance in the form of IQ, parental tobacco smoking/alcohol consumption, ethnicity (including African, Caucasian, Asian, and Hispanic), parental history of ADHD, geographical latitude of where the study was conducted, and the type of assay used to detect the iron level.

### Assessment of study quality

We used the Newcastle-Ottawa Scale for cohort studies and case control studies. For cross-sectional studies, we used a modified version of the Newcastle-Ottawa Scale for observational studies to assess the quality of the included studies. This modified version of the Newcastle-Ottawa Scale score for observational studies ranges from zero to six, and a score greater than three was classified as a high-quality study. For case control studies, the Newcastle-Ottawa Scale score ranges from zero to ten, with a score greater than five being classified as a high-quality study^41^. For cohort studies, the Newcastle-Ottawa Scale score ranges from zero to nine, with a score of six or more being classified as a high-quality study^40^.

### Statistical analysis

The current study was conducted in two parts. First, we analyzed the data considering iron in relation to the children with ADHD compared to the controls. Second, we analyzed the data about the severity of ADHD symptoms or OR of ADHD in the children with ID compared to the controls. To control for the potential confounding effects of clinical variables, we performed further meta-analysis based on the pooled adjusted OR from the recruited studies. In brief, we extracted the adjusted OR with regards to the association of ADHD in the children with ID from the recruited studies to calculate the pooled adjusted OR of the association between ADHD and ID.

Based on the presumed heterogeneity of background and population among the recruited studies, we conducted the meta-analyses with a random effects model rather than a fixed effects model^57^. In brief, random-effects modeling is more stringent than fixed-effects modeling and incorporates a between-study variance in the calculations^58^. For continuous outcomes (i.e. differences in iron levels between the children with ADHD and the controls), we calculated Hedges’ *g* and 95% CI. For dichotomous outcomes (i.e. differences in the prevalence of ID between the children with ADHD and the controls) we calculated the OR and 95% CI.

### Heterogeneity, publication bias, and sensitivity test

Heterogeneity was assessed using the Cochran Q test^59^. The *I*^2^ statistic should be interpreted as the proportion of heterogeneity a study estimates that is due to heterogeneity^60^. For publication bias, we used direct inspection of funnel plots for fewer than 10 datasets^61^ and Egger’s regression test for 10 or more datasets^62^. We also used the Duval and Tweedie’s trim-and-fill procedure to adjust the ESs when publication bias was evident^63^. We also used a sensitivity test with one study removed to investigate any potential outliers present in the recruited studies^64^.

### Subgroup meta-analysis and meta-regression analysis

To discover any potential sources of heterogeneity, subgroup analyses were performed to explore potential interactions between clinical variables and peripheral iron status parameters in the children with ADHD compared to the controls. We only performed subgroup analysis whenever data from three independent datasets were available^65^. The main clinical target for subgrouping included sample sources, subjects who were or were not drug free, and the situation when blood was drawn. When data were available for a moderator in more than five studies, we performed unrestricted maximum likelihood random-effects meta-regression to explore any potential source of heterogeneity. The moderators of interest included mean age, female proportion, body mass index, percentage of ADHD subtypes, mean IQ, percentage of parental tobacco smoking/alcohol consumption, percentage of each ethnicity, percentage of parental history of ADHD, sample size of the disease groups, and geographical latitude of where the study was conducted.

The meta-analyses were conducted using Comprehensive Meta-Analysis software, version 3 (Biostat, Englewood, NJ).

### Data availability

The datasets generated and analyzed during the current study are available from the corresponding author on reasonable request.

## Electronic supplementary material

Supplementary information
