# Supplementary material for: Peripheral iron levels in children with attention-deficit hyperactivity disorder: a systematic review and meta-analysis
Source: Sci Rep. 2018 Jan 15;8:788. doi: 10.1038/s41598-017-19096-x (PMC5768671; doi:10.1038/s41598-017-19096-x)
Supplement: Supplementary file 1 — Supplementary information [file 41598_2017_19096_MOESM1_ESM.pdf]

Peripheral iron levels in children with attention-deficit hyperactivity disorder: a systemic review and meta-analysis

Ping-Tao Tseng, Yu-Shian Cheng, Cheng-Fang Yen, Yen-Wen Chen, Brendon Stubbs, Paul Whiteley, Andre F Carvalho, Dian-Jeng Li, Tien-Yu Chen, Wei-Cheng Yang, Chia-Hung Tang, Che-Sheng Chu, Wei-Chieh Yang, Hsin-Yi Liang, Ching-Kuan Wu, Pao-Yen Lin

Supplementary figure 1: Detailed search results from all databases

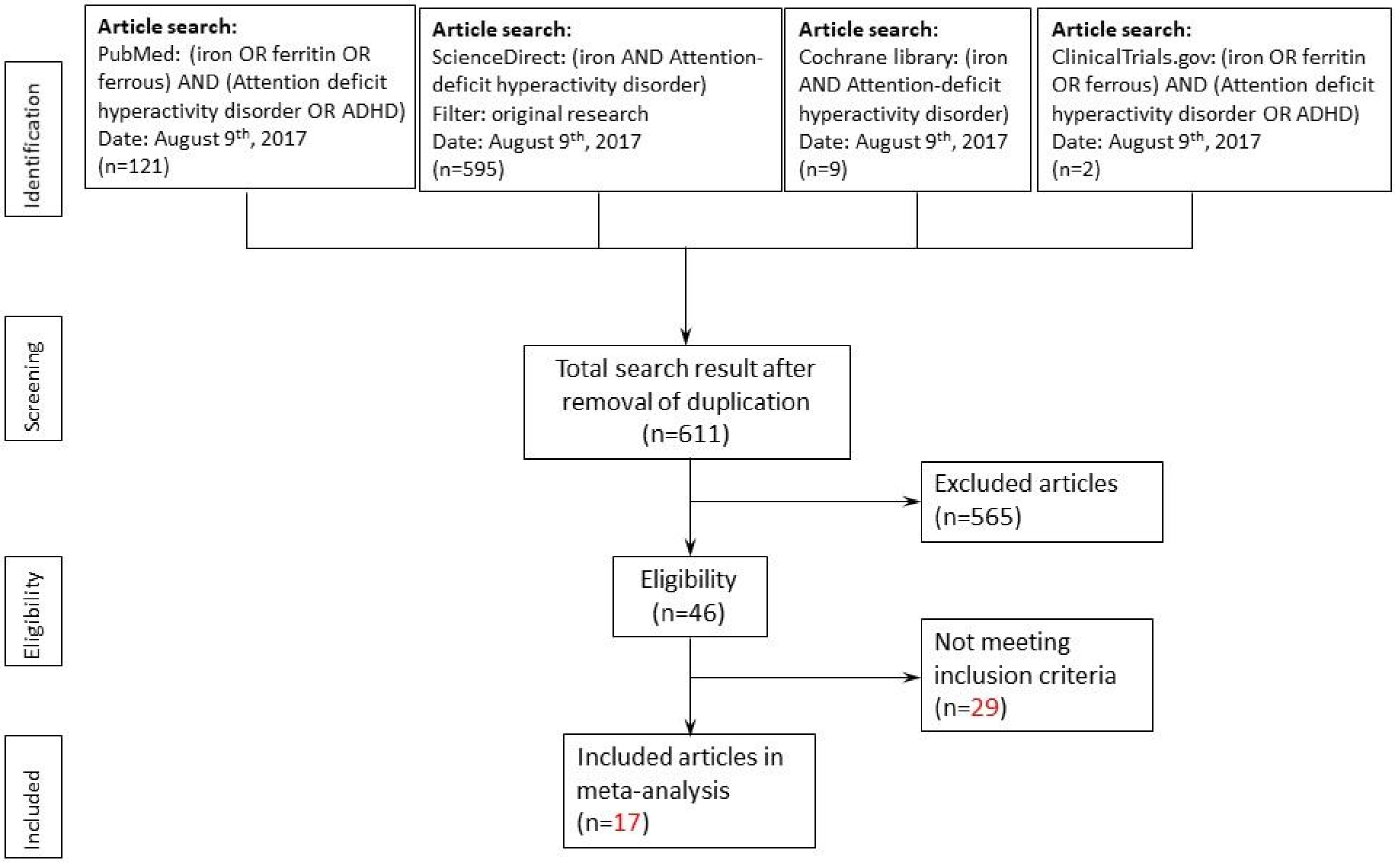

Supplement figure 1 Detailed search result from each databases

Supplementary figure 2A Funnel plot of MA of comparison of serum iron levels in children with/without ADHD

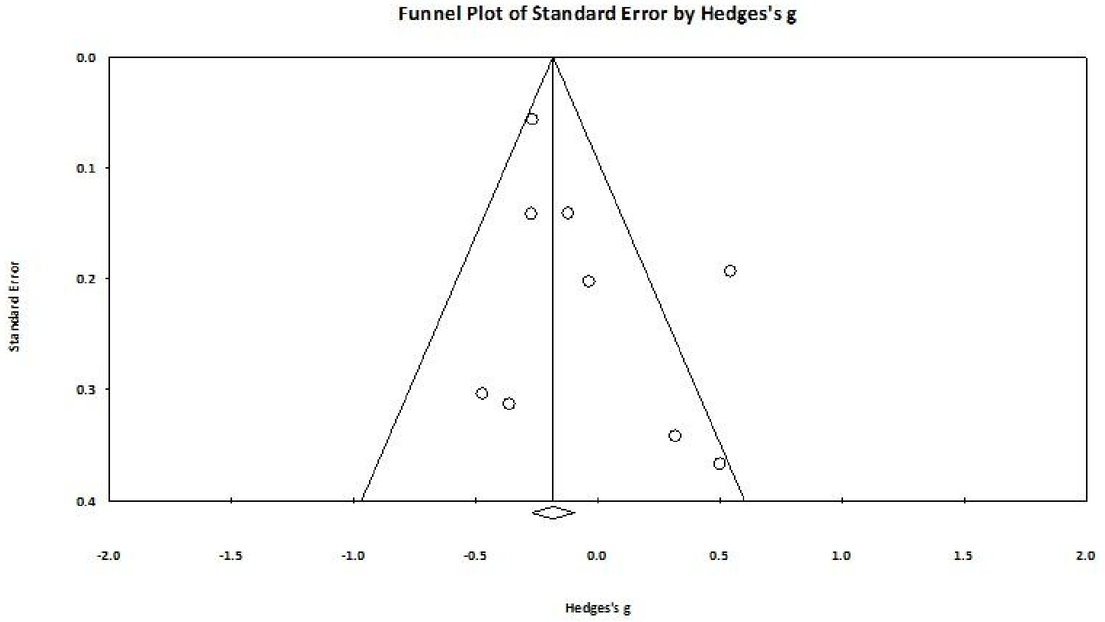

Supplement figure 2A Funnel plot of MA of comparison of serum iron levels in children with/without ADHD

Supplementary figure 2B Funnel plot of MA of comparison of serum transferrin levels in children with/without ADHD

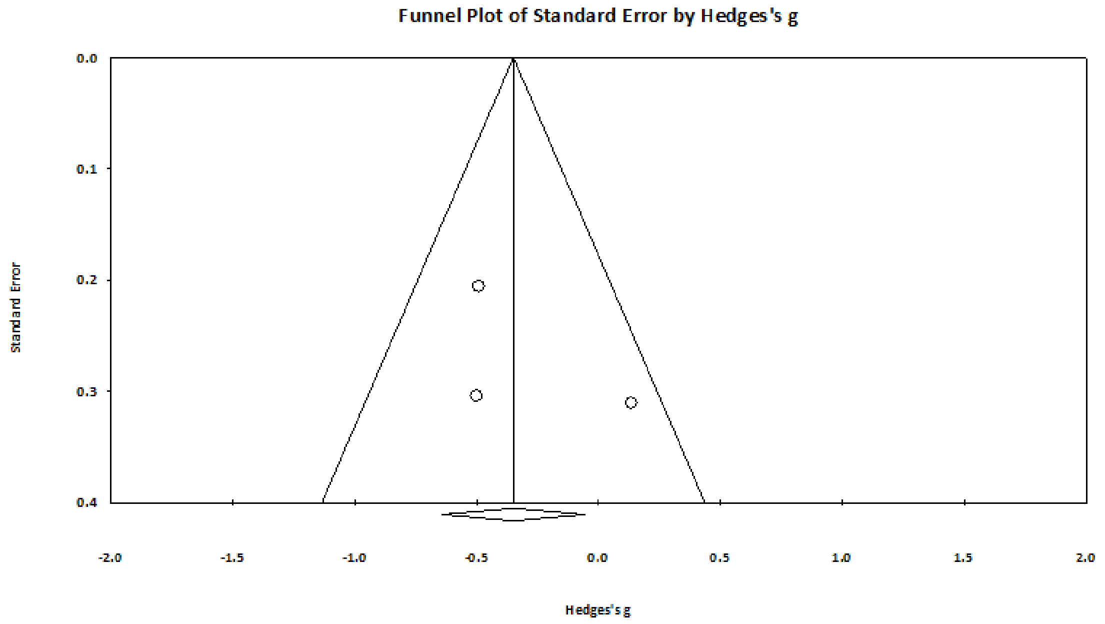

Supplement figure 2B Funnel plot of MA of comparison of serum transferrin levels in children with/without ADHD

Supplementary figure 2C Funnel plot of MA of comparison of ADHD severity in children with/without ID

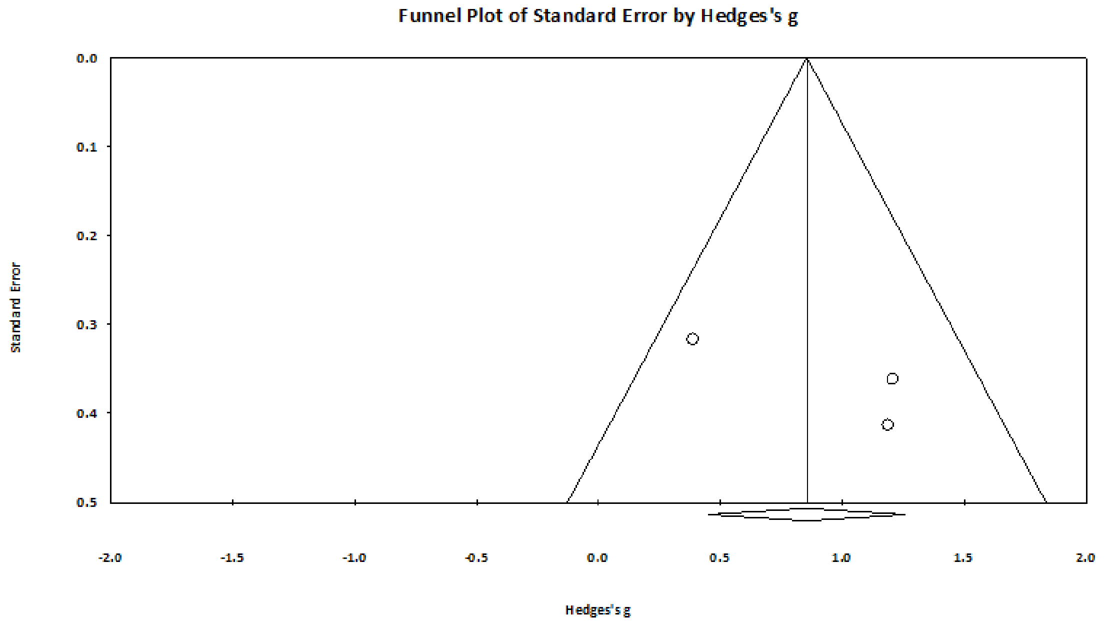

Supplement figure 2C Funnel plot of MA of comparison of ADHD severity in children with/without ID

Supplementary figure 2D Funnel plot of MA of pooled adjusted OR of association between ADHD and ID

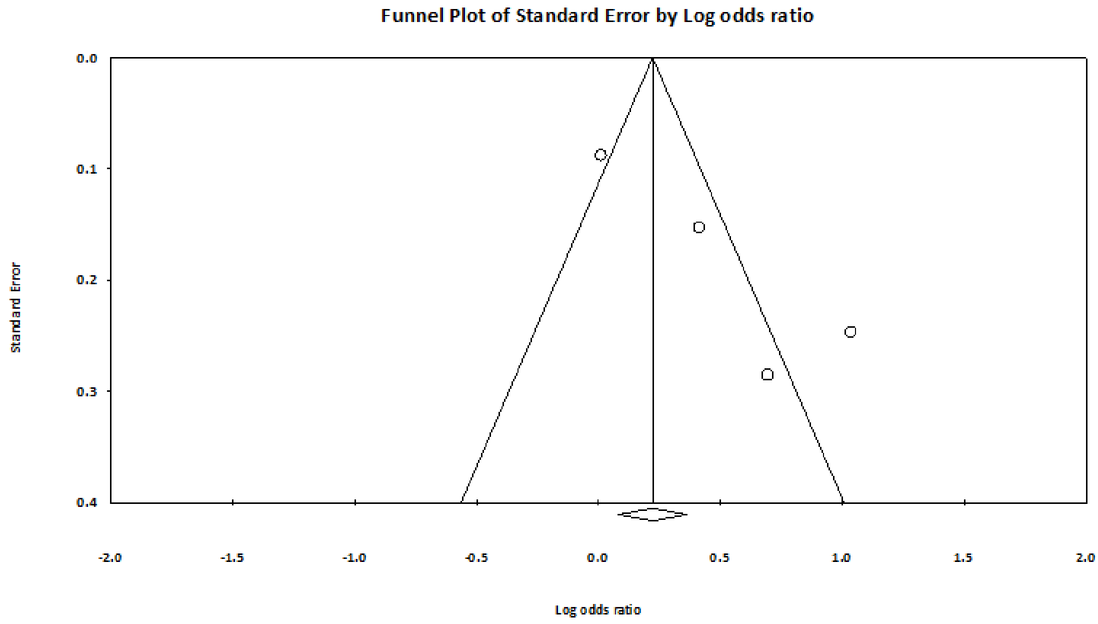

Supplement figure 2D Funnel plot of MA of pooled adjusted OR of association between ADHD and ID

Supplementary table 1: MOOSE Checklist for Meta-analyses of Observational Studies

Item No	Recommendation	Reported on Page No
Reporting of background should include		
1	Problem definition	5-7
2	Hypothesis statement	5-7
3	Description of study outcome(s)	5-7
4	Type of exposure or intervention used	N/A
5	Type of study designs used	5-7
6	Study population	5-7
Reporting of search strategy should include		
7	Qualifications of searchers (eg, librarians and investigators)	18-19
8	Search strategy, including time period included in the synthesis and key words	18-19
9	Effort to include all available studies, including contact with authors	18-20
10	Databases and registries searched	18-20
11	Search software used, name and version, including special features used (eg, explosion)	18-20
12	Use of hand searching (eg, reference lists of obtained articles)	19-20
13	List of citations located and those excluded, including justification	20-22
14	Method of addressing articles published in languages other than English	18-20
15	Method of handling abstracts and unpublished studies	18-19
16	Description of any contact with authors	19-20
Reporting of methods should include		
17	Description of relevance or appropriateness of studies assembled for assessing the hypothesis to be tested	19-22
18	Rationale for the selection and coding of data (eg, sound clinical principles or convenience)	19-22
19	Documentation of how data were classified and coded (eg, multiple raters, blinding and interrater reliability)	20-22
20	Assessment of confounding (eg, comparability of cases and controls in studies where appropriate)	19-20
21	Assessment of study quality, including blinding of quality assessors, stratification or regression on possible predictors of study results	19-20
22	Assessment of heterogeneity	20-22
23	Description of statistical methods (eg, complete description of fixed or random effects models, justification of whether the chosen models account for predictors of study results, dose-response models, or cumulative meta-analysis) in sufficient detail to be replicated	20-22
24	Provision of appropriate tables and graphics	table 1 figure 2
Reporting of results should include		
25	Graphic summarizing individual study estimates and overall estimate	figure 2
26	Table giving descriptive information for each study included	table 1
27	Results of sensitivity testing (eg, subgroup analysis)	N/A
28	Indication of statistical uncertainty of findings	N/A

Item No	Recommendation	Reported on Page No
Reporting of discussion should include		
29	Quantitative assessment of bias (eg, publication bias)	8-12
30	Justification for exclusion (eg, exclusion of non-English language citations)	8-12
31	Assessment of quality of included studies	8-12
Reporting of conclusions should include		
32	Consideration of alternative explanations for observed results	17
33	Generalization of the conclusions (ie, appropriate for the data presented and within the domain of the literature review)	17
34	Guidelines for future research	17
35	Disclosure of funding source	23

From: Stroup DF, Berlin JA, Morton SC, et al, for the Meta-analysis Of Observational Studies in Epidemiology (MOOSE) Group. Meta-analysis of Observational Studies in Epidemiology. A Proposal for Reporting. *JAMA*. 2000;283(15):2008-2012. doi: 10.1001/jama.283.15.2008.

Transcribed from the original paper within the NEUROSURGERY® Editorial Office, Atlanta, GA, United Sates. August 2012.

Supplementary table 2: Excluded studies and reasons

Not comparing iron in ADHD and controls (n=5)

Yehuda, S., S. Rabinovitz-Shenkar, and R.L. Carasso, *Effects of essential fatty acids in iron deficient and sleep-disturbed attention deficit hyperactivity disorder (ADHD) children.* Eur J Clin Nutr, 2011. **65**(10): p. 1167-9.

Calarge, C.A., et al., *Iron homeostasis during risperidone treatment in children and adolescents.* J Clin Psychiatry, 2015. **76**(11): p. 1500-5.

Elbaz, F., S. Zahra, and H. Hanafy, *Magnesium, zinc and copper estimation in children with attention deficit hyperactivity disorder (ADHD).* The Egyptian Journal of Medical Human Genetics, 2016.

Ode, A., et al., *Manganese and selenium concentrations in umbilical cord serum and attention deficit hyperactivity disorder in childhood.* Environ Res, 2015. **137**: p. 373-81.

Gottfried, R.J., et al., *The iron status of children and youth in a community mental health clinic is lower than that of a national sample.* J Child Adolesc Psychopharmacol, 2013. **23**(2): p. 91-100.

Lack of controls (n=8)

Oner, P. and O. Oner, *Relationship of ferritin to symptom ratings children with attention deficit hyperactivity disorder: effect of comorbidity.* Child Psychiatry Hum Dev, 2008. **39**(3): p. 323-30.

Oner, P., et al., *Association between low serum ferritin and restless legs syndrome in patients with attention deficit hyperactivity disorder.* Tohoku J Exp Med, 2007. **213**(3): p. 269-76.

Oner, O., et al., *Effects of zinc and ferritin levels on parent and teacher reported symptom scores in attention deficit hyperactivity disorder.* Child Psychiatry Hum Dev, 2010. **41**(4): p. 441-7.

Oner, P., et al., *Ferritin and hyperactivity ratings in attention deficit hyperactivity disorder.* Pediatr Int, 2012. **54**(5): p. 688-92.

Oner, O., O.Y. Alkar, and P. Oner, *Relation of ferritin levels with symptom ratings and cognitive performance in children with attention deficit-hyperactivity disorder.* Pediatr Int, 2008. **50**(1): p. 40-4.

Cortese, S., et al., *Sleep disturbances and serum ferritin levels in children with attention-deficit/hyperactivity disorder.* Eur Child Adolesc Psychiatry, 2009. **18**(7): p. 393-9.

Miano, S., et al., *Sleep-Related Disorders in Children with Attention-Deficit Hyperactivity Disorder: Preliminary Results of a Full Sleep Assessment Study.* CNS Neurosci Ther, 2016.

Lahat, E., et al., *Iron deficiency in children with attention deficit hyperactivity disorder*. Isr Med Assoc J, 2011

Commentary articles (n=8)

Cortese, S., et al., *Attention-deficit/hyperactivity disorder, Tourette's syndrome, and restless legs syndrome: the iron hypothesis.* Med Hypotheses, 2008. **70**(6): p. 1128-32.

Bellinger, D.C., *Comparing the population neurodevelopmental burdens associated with children's exposures to environmental chemicals and other risk factors.* Neurotoxicology, 2012. **33**(4): p. 641-3.

Parisi, P., et al., *Could treatment of iron deficiency both improve ADHD and reduce cardiovascular risk during treatment with ADHD drugs?* Med Hypotheses, 2012. **79**(2): p. 246-9.

Picchietti, D., *Is iron deficiency an underlying cause of pediatric restless legs syndrome and of attention-deficit/hyperactivity disorder?* Sleep Med, 2007. **8**(7-8): p. 693-4.

Konofal, E. and S. Cortese, *Lead and neuroprotection by iron in ADHD.* Environ Health Perspect, 2007. **115**(8): p. A398-9; author reply A399.

Ghanizad, A., *Regarding the effects of iron supplementation on attention deficit hyperactivity disorder in children.* Pediatr Neurol, 2008. **39**(1): p. 73; author reply 73-4.

Poulton, A., *To the editor: efficacy of oral iron for the treatment of attention deficit hyperactivity disorder in children with low ferritin levels.* Pediatr Neurol, 2008. **39**(1): p. 74; author reply 74.

Sabuncuoglu, O., *Understanding the relationships between breastfeeding, malocclusion, ADHD, sleep-disordered breathing and traumatic dental injuries.* Med Hypotheses, 2013. **80**(3): p. 315-20.

Review articles (n=3)

Scassellati, C., et al., *Biomarkers and attention-deficit/hyperactivity disorder: a systematic review and meta-analyses.* J Am Acad Child Adolesc Psychiatry, 2012. **51**(10): p. 1003-1019 e20.

Adisetiyo, V. and J.A. Helpern, *Brain iron: a promising noninvasive biomarker of attention-deficit/hyperactivity disorder that warrants further investigation.* Biomark Med, 2015. **9**(5): p. 403-6.

Yui, K., et al., *Eicosanoids Derived From Arachidonic Acid and Their Family Prostaglandins and Cyclooxygenase in Psychiatric Disorders.* Curr Neuropharmacol, 2015. **13**(6): p. 776-85.

<u>Not related to the specific treatment of iron supplementary therapy (n=3)</u>

Rucklidge, J.J., et al., *Moderators of treatment response in adults with ADHD treated with a vitamin-mineral supplement.* Prog Neuropsychopharmacol Biol Psychiatry, 2014. **50**: p. 163-71.

Calarge, C., et al., *Serum ferritin and amphetamine response in youth with attention-deficit/hyperactivity disorder.* J Child Adolesc Psychopharmacol, 2010. **20**(6): p. 495-502.

Arnold, L.E., et al., *Zinc for attention-deficit/hyperactivity disorder: placebo-controlled double-blind pilot trial alone and combined with amphetamine.* J Child Adolesc Psychopharmacol, 2011. **21**(1): p. 1-19.

<u>Full article not available (n=1)</u>

Kotchabhakdi, N. and K. N.J., *S18.D ADHD, RLS, and PLMD in Thai children and their links to iron deficiency.* Sleep Medicine, 2007. **Suppl 1**: p. S11–S47.

<u>Compared iron hemoglobin but not iron, ferritin, or transferrin levels (n=1)</u>

Nigg, J.T., et al., *Variation in an Iron Metabolism Gene Moderates the Association Between Blood Lead Levels and Attention-Deficit/Hyperactivity Disorder in Children.* Psychol Sci, 2016. **27**(2): p. 257-69.

Supplementary table 3: Newcastle-Ottawa Scale for observational studies

Author (year)	Country	Design	Selection	Comparability	Outcome	Total
Subgroup meta-analysis of comparison of the peripheral iron levels in children with ADHD and those without ADHD (Study numbers=17)						
Bala, K.A. (2016)	Turkey	Case control study	4	0	3	7(high quality)
Percinel, I. (2016)	Turkey	Case control study	3	0	3	6(high quality)
Adisetiyo, V. (2014)	USA	Cross-sectional study	2	1	2	5(high quality)
Bener, A. (2014)	Qatar	Case control study	4	2	3	9(high quality)
Donfrancesco, R. (2013)	Italy	Case control study	4	1	3	8(high quality)
Romanos, M. (2013)	Germany	Cross-sectional study	2	2	1	5(high quality)
Cortese, S. (2012)	France	Case control study	3	2	3	8(high quality)
Kwon, H.J. (2011)	Korea	Case control study	4	0	3	7(high quality)
Juneja, M. (2010)	India	Case control study	3	1	3	7(high quality)
Menegassi, M.(2010)	Brazil	Case control study	2	0	4	6(high quality)

Konofal, E. (2007)	France	Case control study	3	0	3	6(high quality)
Millichap, J.G. (2006)	USA	Case control study	3	0	3	6(high quality)
Chen J.R. (2004)	Taiwan	Case control study	2	0	3	5(low quality)
Konofal, E. (2004)	France	Cross-sectional study	2	1	1	4(high quality)
Subgroup meta-analysis of comparison of the symptoms severity of ADHD in children with iron deficiency and those without iron deficiency (Study numbers=6)						
Doom, J.R. (2015)	USA	Cohort study	2	0	2	4(low quality)
Bener, A. (2014)	Qatar	Cross-sectional study	4	2	3	9(high quality)
Abou-Khadra, M.K. (2013)	Egypt	Cross-sectional study	2	0	1	3(low quality)
Chen M.H. (2013)	Taiwan	Cross-sectional study	2	2	2	6(high quality)
Fuglestad, A.J. (2013)	USA	Cohort study	3	2	1	6(high quality)
Romanos, M. (2013)	Germany	Cross-sectional study	2	2	1	5(high quality)

We used the Newcastle-Ottawa Scale for cohort studies and case control studies. For cross sectional studies, we used modified version of the Newcastle-Ottawa Scale for observational studies to assess the quality of included studies. For cross-sectional studies, the modified version of the Newcastle-Ottawa Scale for observational studies score ranges from zero to six and score greater than three was classified as high quality studies [1]. For case control studies, the Newcastle-Ottawa Scale ranges

from zero to ten and score greater than five was classified as high quality studies[1]. For cohort studies, the Newcastle-Ottawa Scale for cohort studies score ranges from zero to nine and score of six or more was classified as high quality studies [2].

1. Juni, P., et al., *The hazards of scoring the quality of clinical trials for meta-analysis.* Jama, 1999. **282**(11): p. 1054-60.
2. Stang, A., *Critical evaluation of the Newcastle-Ottawa scale for the assessment of the quality of nonrandomized studies in meta-analyses.* Eur J Epidemiol, 2010. **25**(9): p. 603-5.

Supplementary table 4: Meta-regression of primary outcome and clinical variables

Clinical variables	slope	*p* value
Peripheral ferritin levels in ADHD children		
Mean age	0.187	*0.025
Proportion of female	0.020	0.061
Percentage of combined subtype of ADHD	-0.004	0.550
Percentage of inattention subtype of ADHD	-0.001	0.860
Percentage of hyperactivity/impulsivity subtype of ADHD	0.001	0.884
Cognition (in forms of IQ)	0.029	0.574
Sample size of ADHD groups	0.001	0.740
Latitude of where the study was conducted	-0.006	0.690
Peripheral iron levels in ADHD children		
Mean age	0.015	0.827
Proportion of female	0.001	0.958
Sample size of ID groups	-0.001	0.388
Latitude of where the study was conducted	0.011	0.361

*: indicated *p* value < 0.05
